# Bone growth and metabolism in broilers exposed to dietary PUFA or cyclical heat stress under two levels of dietary vitamins and minerals

**DOI:** 10.3389/fphys.2026.1771630

**Published:** 2026-07-03

**Authors:** Jakob Leskovec, Alenka Levart, Vida Rezar, Tatjana Pirman, Janez Salobir, Milka Vrecl, Jana Brankovič

**Affiliations:** 1 Department of Animal Science, University of Ljubljana Biotechnical Faculty, Domžale, Slovenia; 2 Institute for Food and Agricultural Research and Technology (IRTA), Animal Nutrition, Constantí, Tarragona, Spain; 3 Institute of Preclinical Sciences, University of Ljubljana Veterinary Faculty, Ljubljana, Slovenia

**Keywords:** antioxidant, bone growth and metabolism, broiler, heat stress, polyunsaturated fatty acid

## Abstract

Broilers are highly susceptible to heat stress (HS), which can impair bone growth and metabolism. Supplementation with n-3 polyunsaturated fatty acids (PUFA) may affect the metabolism of mineralized tissues, whereas antioxidants can mitigate these effects. The aim was to investigate the combined effects of HS (thermoneutral conditions vs. cyclic HS) or dietary oxidative stress induced by supranutritional n-3 PUFA (control feed without PUFA vs. feed supplemented with 5% linseed oil) with two levels (lower and higher) of dietary antioxidants and minerals on bone properties. The morphology and geometry of the proximal tibiotarsal growth plate, biomechanical behavior, and mineral composition of 42-day-old male Ross 308 broilers were evaluated. HS and high PUFA levels decreased bone size and mass, whereas supplementation with high antioxidant levels had the opposite effect. High PUFA supplementation induced histopathological changes in the growth plate that resembled tibial dyshondroplasia; these effects were attenuated by supplementation with high levels of antioxidants. Additionally, HS had a negative effect on the biomechanics and reduced the crude ash content. High levels of antioxidants increased the bone ash content, fat content, Ca content and Ca:P ratio. Our results suggest that HS and PUFA impair the growth of bones, hindering the overall growth of fast-growing broilers. The incidence of dyshondroplasia appears to be related to diet rather than to environmental changes. High antioxidant levels mitigate the negative effects of both stressors to some extent, suggesting that antioxidants have positive effects on bone growth and functionality, especially under nonoptimal environmental conditions and dietary stress.

## Introduction

Modern broilers are highly susceptible to environmental changes because of their genetics and strict nutritional and environmental requirements. Studies have reported negative effects of high ambient temperatures, i.e., cyclic heat stress (HS), on tibiotarsus geometry (decreased length, width and mass), contents (decreased ash, Ca and P contents) and biomechanics (decreased strength) in male and female broilers (Vakili et al., 2010; Campos et al., 2016; Hosseini-Vashan et al., 2016; Rocchi et al., 2022). Dietary supplementation with n-3 polyunsaturated fatty acids (PUFA) improved bone strength at lower doses (Ebeid et al., 2011; Ghobashy et al., 2023) and suggested favorable effects on skeletal integrity in broiler offspring when included in the maternal diet (Tompkins et al., 2022). However, higher PUFA doses induced oxidative stress (Voljc et al., 2011), affected mineralized tissue metabolism (Wauquier et al., 2009) and reduced tibiotarsus strength and size in female broilers (Vakili et al., 2010). Various strategies have been proposed to mitigate the negative effects of HS and high PUFA levels (Voljc et al., 2011; Hosseini-Vashan et al., 2016; Al-Sultan et al., 2019). Supplementation with vitamins E, C, Zn, and Se improved bone size, ash content, and mineral density in female and male broilers (Vakili et al., 2010; Calik et al., 2022).

Bone deformities and disorders cause major economic losses in the poultry industry (Knowles et al., 2008; Liu et al., 2023; Szmek et al., 2025). In fast-growing broiler breeds, altered bone metabolism and impaired ossification, especially in long bones of the hind limbs, compromise health and welfare of these animals (Knowles et al., 2008). Compared with slower-growing strains, their bones are less mineralized and more porous and have a higher Ca/P ratio, resulting in altered biomechanical properties (Williams et al., 2000, Williams et al., 2004). Additionally, tibial dyschondroplasia (TD), a common skeletal disorder in rapidly growing broilers, leads to bone deformities and lameness (Leach and Gay, 1987). Its incidence, i.e., the number of observed macroscopic TD lesions, was reported to be 24.22% in Ross 308 and Cobb 500 and 27.7% in Pureline broilers (Dinev et al., 2012). A recent study revealed a 22% incidence in yellow-feathered Chinese meat chickens at 6 weeks, which decreased after 8 weeks (Shi et al., 2024). Body weight, tibial length and quality parameters (mineral content and density, ash content, calcium content, and phosphorus content) were decreased in broilers in the TD group compared with those in the control group (Shi et al., 2024). Its etiology involves dietary, environmental, and genetic factors (Rennie et al., 1993; Jahejo and Tian, 2021), whereas hypoxia may increase vascular density and proximal tibial EGP thickness (Huang et al., 2017).

Since bone tissue can limit animal growth, understanding the effects of environmental and nutritional stressors on bone tissue is of paramount importance.

This study examined the effects of cyclic HS or dietary oxidative stress induced by supranutritional n-3 PUFA, combined with two dietary regimens of antioxidants and minerals: (i) commercial recommendations (Aviagen, 2019) supplemented with vitamin E, C, and Se, and (ii) National Research Council (NRC) recommendations (Council, N.R 1994), on the bone characteristics and metabolism of male broilers aged 0–42 days during active EGP. To our knowledge, this is the first study to comprehensively evaluate these combined stressors with antioxidant supplementation on bone characteristics in fast-growing broilers complementing previous work on oxidative stress and gut physiology (Rezar et al., 2024, Rezar et al., 2025). In those studies high n-3 PUFA levels reduced body weight, increased relative organ weight, induced oxidative stress and damaged intestinal morphology, whereas antioxidant supplementation increased body weight but reduced relative organ weight (Rezar et al., 2024).

## Materials and methods

### Study design

The study was conducted at the Department of Animal Science, Biotechnical Faculty, University of Ljubljana, Slovenia, and was approved by the Administration for Food Safety, Veterinary Sector and Plant Protection (U34401-5/2021/4), which confirmed that the animal procedures complied with Slovenian and EU regulations. Animal care and procedures were performed in accordance with the European Directive 2010/63/EU, the ARRIVE 2.0 guidelines (Percie du Sert et al., 2020) and the PREPARE guidelines for the design of animal research and experimentation (Smith et al., 2018).

One-day-old male Ross 308 broilers, n = 192 (individually labeled on day 1), were divided into groups according to a 2 × 2 × 2 factorial design (**Table 1**). The animals were housed in 24 floor pens measuring 0.95 m × 1.26 m each (with a stock density of 7 animals/m^2^ and 17 kg/m^2^ at the end of the study). The animals were reared on wood shavings (approx. 10 cm in depth), with *ad libitum* access to feed and water, and were fed according to a commercial feeding schedule with 3 phases (starter, grower, and finisher; **Table 2**). From day 22 to the end of the trial, the animals were maintained in two different rooms under either thermoneutral (TN, room 1, following Ross recommendations; up to 3 days 32 °C and then gradually lowering it to 21 °C from day 21 onward) or HS conditions (room 2, 12 hours at 24 °C ± 0.5 °C, 2 hours of warming from 24 °C to 34 °C ± 0.5 °C, 7 hours at 34 °C ± 0.5 °C, and 3 hours of cool-down from 34 °C to 24 °C ± 0.5 °C). In each room, 96 animals were housed in 12 pens (8 animals/pen), which were divided into 4 different nutritional groups. Each nutritional group consisted of 24 animals housed in 3 pens. Under each temperature regime, the feeds were based on two different fat sources: a 5% mixture of vegetal and animal fats with low PUFA content (low PUFA; LP) or 5% cold-pressed linseed oil with high PUFA content (high PUFA, HP) and two levels of antioxidants and minerals according to NRC requirements (Council, N.R 1994) (low antioxidant content; LA) or high levels of antioxidants according to commercial recommendations by Ross (Aviagen, 2019); the latter diets were supplemented with 200 IU vitamin E/kg, 250 mg vitamin C/kg and 0.15 mg organic Se/kg feed (high antioxidant content; HA). In summary, the animals were fed 4 different diets (LP/LA, LP/HA, HP/LA, and HP/HA) under two different temperature regimes (**Table 1**).

**Table 1:** Study design.

Study design (n = 192)								
Environment	TN (n = 96)				HS (n = 96)			
Diet - PUFA	Low (n = 48) - **LP**		High (n = 48) - **HP**		Low (n = 48) - **LP**		High (n = 48) - **HP**	
Diet – mineral and antioxidant levels	Low 3 pens; n = 24 **LA**	High 3 pens; n = 24 **HA**	Low 3 pens; n = 24 **LA**	High 3 pens; n = 24 **HA**	Low 3 pens; n = 24 **LA**	High 3 pens; n = 24 **HA**	Low 3 pens; n = 24 **LA**	High 3 pens; n = 24 **HA**

TN, thermoneutral environment; HS, heat stress environment; PUFA, polyunsaturated fatty acids. Nomenclature of the experimental diets per experimental group: LP/LA, no supplementation with an additional fat source, basal diet according to the NRC; HP/LA, basal diet +5% linseed oil and NRC; LP/HA, no supplementation with an additional fat source, Aviagen + 200 IU dl-α-tocopheryl acetate + 250 mg vitamin C + 0.15 mg Se/kg feed; HP/HA, basal diet +5% linseed oil and Aviagen + 200 IU dl-α-tocopheryl acetate + 250 mg vitamin C + 0.15 mg Se/kg feed.

The numbers of animals in each group are in parentheses. LP, low dietary PUFA; HP, high dietary PUFA (5% linseed oil); LA, NRC requirements for vitamins and minerals; HA, Aviagen requierements for vitamins and minerals + 200 IU dl-α-tocopheryl acetate + 250 mg vitamin C + 0.15 mg Se/kg feed.

**Table 2:** Composition and calculated nutrient content of the starter, grower and finisher diets.

	Starter	Grower	Finisher
Composition, %			
Maize	30.98	37.99	52.68
Wheat	14.0	13.0	4.0
Wheat flour	3.00	1.50	0.00
Soya meal	33.2	28.0	24.0
Corn gluten meal	8.60	9.30	9.41
Fat	5.40	5.72	5.72
Salt	0.0491	0.0495	0.0505
Monocalcium phospate	0.171	0.158	0.145
Limestone	0.145	0.124	0.122
L-lysine-HCl	0.35	0.34	0.33
DL-methionine	0.022	0.018	0.015
L-threonine	0.008	0.005	0.003
Mineral-vitamin premix	0.05	0.05	0.05
Calculated			
Metabolizable energy, MJ/kg	12.42	12.82	13.20
Crude protein, %	24.6	22.8	20.8
Lysine, %	1.28	1.15	1.03
Calcium, %	0.96	0.88	0.79
Available phosphorus, %	0.48	0.44	0.40

A detailed description of the feed is available in (Rezar et al., 2025).

The animals (12 randomly selected chickens per experimental group; 3 per pen) were weighed and killed on day 42 by percussive blow to the head and subsequent exsanguination. Different organs (heart, liver, pancreas, proventriculus, gizzard, small intestine, cecum) and breast muscle were harvested from each chicken at different “sample-collecting-stations” with a knife or a scalpel, and the left and right tibiotarsi were removed. Each person harvested one type of organ from all the euthanized animals. The left and right hind legs were removed from the body at the hip joint (*articulatio coxae*), and both tibiotarsi (with menisci) were separated from the femurs and tarsometatarsi at the knee (*articulatio femorotibialis*) and the ankle joint (*articulatio intertarsales*), respectively, with a kitchen knife. After disarticulation, soft tissues (muscles, tendons, ligaments), menisci and fibulas were removed, and the tibiotarsi were cleaned with scissors and a scalpel. The bones were cleaned of several muscles and their tendons (*mm. gastrocnemius, peroneus longus, tibialis cranialis, flexorii et extensorii*). Relative organ weights (including various parts of the small intestine) and fatty acid profiles of the liver have been analyzed and reported previously (Rezar et al., 2024).

### Feeding and feed composition

The feed mixtures consisted mainly of maize and wheat, with a small percentage of wheat bran in the starter and grower feed. Soybean meal and corn gluten meal were added as protein sources, and synthetic amino acids and feedstuffs were added as sources of macrominerals and 5% premix. The composition, calculated energy and nutrient contents are listed in **Table 2**, and the composition of proximate components, minerals, fatty acids, malondialdehyde and antioxidants in the finisher experimental diets is listed in **Table 3**, additional data on the feed are provided in an article by Rezar et al. (2025).

**Table 3:** Proximate composition, minerals, fatty acids, malondialdehyde, and antioxidants in the finisher experimental diets.

Component	Experimental diets1			
	LP/LA	HP/LA	LP/HA	HP/HA
Dry matter, g/kg	899	899	898	898
Crude protein, g/kg	215	214	214	213
Ether extract, g/kg	84.4	86.7	83.8	84.1
Crude ash, g/kg	55.0	56.7	58.9	54.8
Crude fiber, g/kg	68.4	71.2	68.8	75.1
Nitrogen free extract, g/kg	476	470	472	471
Calcium, g/kg	7.55	7.87	8.41	7.24
Phosphorus	5.91	5.95	6.25	5.94
α-tocopherol, mg/kg	12.2	8.9	250.2	232.2
ACW (µmol/g)	3.37	2.62	2.95	2.51
Selenium, mg/kg	0.18	0.15	0.39	0.35
Fatty acid composition^2^, g of fatty acids/100 g of fatty acids				
C16:0	22.36	8.99	22.09	8.95
C18:0	10.97	3.66	10.66	3.61
C18:2 n-6	26.5	28.4	27.5	28.5
C18:3 n-3	2.08	37.84	2.09	37.77
∑ SFA	37.0	13.4	36.3	13.3
∑ MUFA	34.0	20.4	33.7	20.4
∑ PUFA	29.0	66.2	30.0	66.3
∑ n-6 PUFA	26.8	28.4	27.7	28.5
∑ n-3 PUFA	2.14	37.84	2.16	37.77
n-6/n-3 PUFA	12.53	0.75	12.87	0.76

^1^ Nomenclature of the experimental diets per experimental group: LP/LA, no supplementation with an additional fat source, basal diet according to the NRC; HP/LA, basal diet +5% linseed oil and NRC; LP/HA, no supplementation with an additional fat source, Aviagen + 200 IU dl-α-tocopheryl acetate + 250 mg vitamin C + 0.15 mg Se/kg feed; HP/HA, basal diet +5% linseed oil and Aviagen + 200 IU dl-α-tocopheryl acetate + 250 mg vitamin C + 0.15 mg Se/kg feed. ^2^ Only prevalent and important dietary fatty acids are listed. ACW: antioxidant capacity of water-soluble compounds, SFA: saturated fatty acids, MUFA: monounsaturated fatty acid, PUFA: polyunsaturated fatty acid. Additional information regarding the feed analyses can be found in (Rezar et al., 2025).

The premixes for the feed were calculated according to the NRC mineral and vitamin requirements (Council, N.R 1994) and Ross 308 requirements (Aviagen, 2019) for broilers. The experimental diets were supplemented with the following antioxidant sources: dl-α-tocopheryl acetate (Rovimix E50, DSM, Heerlen, the Netherlands) as a source of vitamin E, vitamin C in the form of Rovimix Stay-C35 (DSM, Heerlen, the Netherlands) and organic Se in the form of SelSaf 3000 (Lesaffre, Marcq-en-Barœul, France). The nutrient composition, concentrations of some minerals, contents of α-tocopherol and γ-tocopherol, malondialdehyde content, antioxidant capacity of water-soluble compounds and fatty acid composition of the experimental diets are presented in the article by Rezar et al. (2024).

### Histomorphometry

The proximal extremity of the left tibiotarsus was cut transversely for histomorphometric analysis using an electric saw with a diamond blade (Bosch Multicutter GOP 40–30 Professional) (Brankovič et al., 2022). After fixation in 10% buffered formalin, the proximal extremities (*n* = 8/group) were decalcified in 10% formic acid (98–100% CH_2_O_2_; Kemika, Zagreb, Croatia) at 21 °C for 72 hours with gentle agitation at 60 strokes/min (Kuhner shaker ISF1-W; Basel, Switzerland) (Gupta et al., 2014). The bone samples were cut sagittally and transversely into four quarters. Macroscopic samples of the proximal extremity of the tibiotarsi were photographed, and “naked eye assessment” of the macroscopic morphology in terms of the extent of EGP was performed as reported previously (Thorp et al., 1991) before the samples were cut into smaller pieces for further decalcification. Changes were graded according to the extent to which the EGP of the proximal extremity was affected (**Figure 1**). No change was graded as 0. Alterations resembling unvascularized, opaque, white cartilage tissue were present medially (grade 1), medially and caudally (grade 2), or throughout the EGP (grade 3). The blood supply in the metaphysis was macroscopically assessed on the basis of its coloration and tissue structure, namely, normal blood supply (yes) and reduced supply (no).

**Figure 1:**
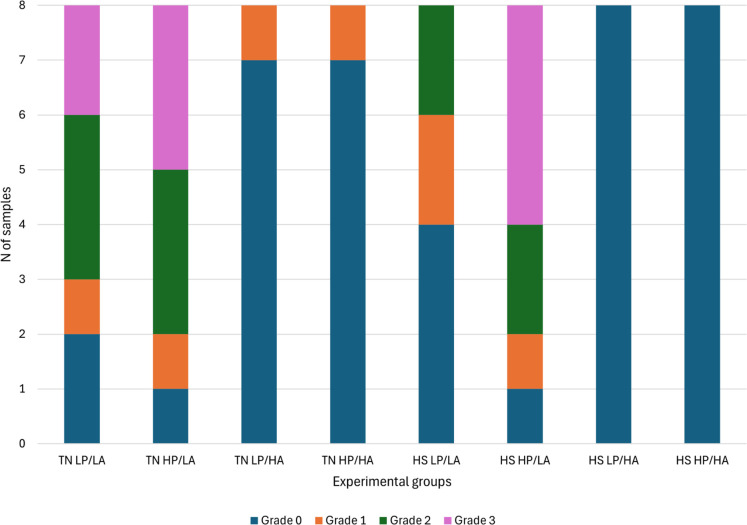
Macroscopic grading of changes in the proximal EGP of the left tibiotarsi of 42-day-old broilers. Scale = 4 mm. Grade 0: no change, zones of the EGP and medullary cavity developed and clearly visible; grade 1: a small part of the EGP affected: enlarged cartilage area extending distally from the proximal EGP toward the medullary cavity on the medial part of the proximal extremity; grade 2: half of the EGP affected: enlarged cartilage area extending distally from the proximal EGP toward the medullary cavity on the medial and caudal part of the proximal extremity; grade 3: most of the EGP affected: enlarged cartilage area extending distally from the proximal EGP toward the medullary cavity throughout the proximal extremity. PZ, proliferative zone; HZ, hypertrophy zone; MC, medullary cavity; UC, unvascularized cartilage; UB, noncalcified bone.

Comparably smaller bone samples were demineralized for 24 hours, washed under running tap water, dehydrated and embedded in paraffin (Tissue Processor Leica, Nussloch, Germany) and Tissue-Tek^®^ TEC™ 5 (Tissue Embedding Console System, Sakura Finetek Europe HQ, Alphen aan den Rijn, the Netherlands). Next, 5-µm tissue sections (Leica SM2000R microtome, Nussloch, Germany) were cut sagittally through the proximal EGP from the center of the extremity, stained, mounted and cover slipped (Gemini AS slide stainer and ClearVue Coverslipper, Thermo Fisher Scientific, Cheshire, United Kingdom) (Brankovič et al., 2022). The following stains were used to assess morphology (Rigueur and Lyons, 2014; Baranowski et al., 2018): hematoxylin and eosin (HE), Alcian blue solution (AB) and Masson-Goldner (Merck, Darmstadt, Germany). Comparable sections from each bone (*n* = 2 consecutive sections/staining) were assessed with a light microscope (Eclipse Ni-U, Nikon, Tokyo, Japan) and a digital camera (DS-Fi1, Nikon, Tokyo, Japan). The main zones of the proximal EGP, i.e., the reserve zone, proliferative zone, hypertrophy zone and calcified cartilage in the ossification zone (RZ, PZ, HZ and CZ, respectively), were morphologically assessed using the NIS Elements BR 4.6 imaging program (Nikon Instruments Europe B.V., Amsterdam, the Netherlands). The thickness of each zone was measured at three locations. HE staining was performed to determine the basic bone structure, AB staining was performed to measure the zone thickness, and Masson–Goldner staining was performed to differentiate calcified tissue from noncalcified tissue (Brankovic et al., 2019; Brankovič et al., 2022). The parameter of EGP alteration was scored on a binary scale, namely, altered (yes) or not altered (no).

### Geometry and biomechanical properties of bones

For the biomechanical experiments, the right tibiotarsi were placed in plastic bags to prevent the bones from drying until all the animals were euthanized. Before the bending test, weight (g, MA12001L/M Precision Balance, Mettler Toledo, Columbus, United States), length, width (mm) and volume (mL) were measured on carefully and precisely cleaned tibiotarsi (*n* = 12/group), as described previously. A calibrated digital sliding caliper (Sylvac S Cal PRO, Lotrič Metrology, Selce, Slovenia) was used to measure the tibiotarsal length from the medial articular surface (*facies articularis medialis*) at the proximal extremity to the distal point of the medial condyle (*condylus medialis*), and its width (minimum and maximum diameters) was measured in the mid-diaphysis (*corpus tibiotarsi*) at 50% of the bone length to the nearest 0.01 mm (Brankovic et al., 2017). Bone volume was measured by Archimedes’ principle of water displacement (Dempster et al., 2013). The relative bone mass was determined by calculating the ratio of bone mass to total body mass at the time of euthanasia. The body mass of the animals, the feed conversion ratio and the effects of the environment and dietary stress on their growth performance have been reported previously (Rezar et al., 2024, Rezar et al., 2025).

Biomechanical properties were determined on fresh bones by a three-point bending test using an Instron universal hydraulic testing machine (Model 3345, 50 kg load cell; Instron Industrial Products, Norwood, MA, USA) immediately after tissue sampling ended, and geometric parameters were measured. The bone was placed horizontally on two rounded supports (diameter of 5 mm, span length of 60 mm); the cranial surface faced upward. Each tibiotarsus was loaded with a press head (diameter of 5 mm) perpendicular to its longitudinal axis at the mid-diaphysis at a loading rate of 50 mm/min until fracture occurred. During the test, the maximum force (N), corresponding flexure extension (mm) and flex modulus (MPa) at the maximum flexure load were recorded. After fracture, the outer and inner minimum and maximum diaphysis diameters were measured at 60% of the bone length to calculate the cortical thickness (Williams et al., 2004; Brankovic et al., 2017).

### Mineral content of bone

To analyze the mineral content of the bone ash, the right tibiotarsi were dried at 80 °C for 20 hours and then at 104 °C for 3 hours (SP50C dryer, Kambič, Semič, Slovenia). After drying, the bone was ashed for 8 hours at 550 °C (L9/11; Nabertherm, Lilienthal, Germany) and then ground in a laboratory mill. The fat content, mineral composition of the ash, dry content (g/kg bone mass) and microelement content (g or mg/kg ash) were determined by standard methods (Naumann et al., 1976). Spectrophotometric determination of P was performed using a Cary 50 Probe spectrophotometer (Varian, Palo Alto, California, USA), and atomic absorption spectroscopy was used for Ca, Mg, Zn, Fe, Na and K with an Aanalyst 200 spectrometer (Pelkin Elmer, Waltham, Massachusetts, USA). The ratio of Ca to P was calculated.

### Statistical analysis

Data on bone geometry, histomorphology, biochemical properties, and mineral content were analyzed using three-way ANOVA with the Proc Glimmix function in SAS software (Ver. 9.4; SAS Institute Inc., Cary, NC, USA). In the statistical model, the environment (house), fat source and antioxidant content and their interaction were considered fixed effects. In addition, the random effect of the pen within the group was used. Least square means (LSMEANS) are presented, and differences were analyzed by a *post hoc* Tukey–Kramer multiple comparison test. The dispersion is expressed as the SEM. The normality of the distribution of the variables was analyzed using the Shapiro–Wilk test. Statistical significance was assumed when *P* ≤ 0.05.

For categorical data and comparisons of proportions, the chi-square test, including Fisher’s exact test, was used, and *P* values were calculated using SPSS software (IBM, version 29.0; NY, USA). If more than 20% of the expected frequencies were less than 5, the assumption was violated, and the frequencies between these groups were not compared.

## Results

### Macroscopic morphology of the proximal EGP of the left tibiotarsus

Macroscopic images of the proximal extremity of the tibiotarsus revealed changes in the architecture of the EGP (grades 1, 2 and 3; **Figure 1**). In grade 3, the zones of the proximal EGP were not sufficiently developed. The number and classification of altered samples in each group are shown in **Figure 2**. The chi-square test demonstrated that the expected frequencies in the eight experimental groups were less than 5; therefore, the frequencies between these groups could not be statistically compared. Macroscopic changes in the EGP (including grades 1–3) were frequent in the HP/LA groups (87.5% of the analyzed samples), and in the LP/LA groups under TN and HS conditions (75% and 50%, respectively). In the HA groups, changes were rarely observed (12.5%) or not at all observed under TN or HS conditions, respectively (**Figure 3**).

**Figure 2:**
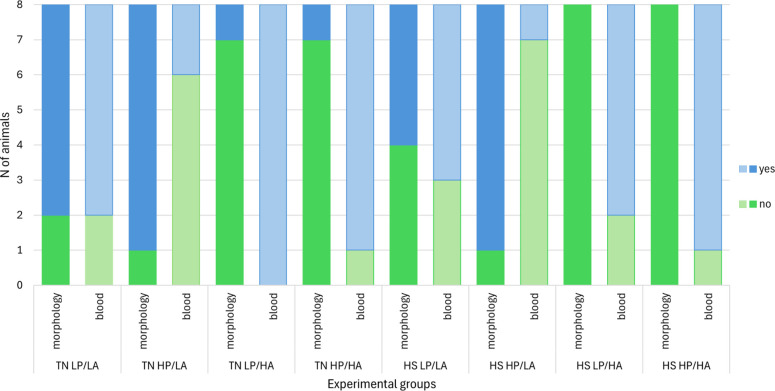
Macroscopic assessment of the proximal EGP of the left tibiotarsi of 42-day-old broilers in four experimental groups reared under two different environmental conditions and fed different experimental diets (*n* = 8/group). Changes were graded according to the extent to which the EGP was affected: no change (grade 0), change present medially (grade 1), change present medially and caudally (grade 2) or in the entire EGP (grade 3). LP/LA: basal diet according to the NRC, no supplementation; HP/LA: +5% linseed oil and NRC; LP/HA: basal diet according to Aviagen + 200 IU dl-α-tocopheryl acetate + 250 mg vitamin C + 0.15 mg Se/kg feed; HP/HA: +5% linseed oil and Aviagen + 200 IU dl-α-tocopheryl acetate + 250 mg vitamin C + 0.15 mg Se/kg feed; TN, thermoneutral environment; HS, heat stress environment.

**Figure 3:**
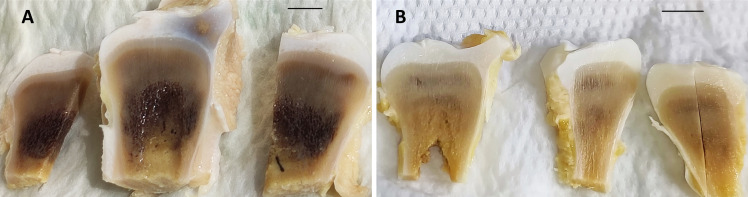
Macroscopic changes in the EGP and blood supply of the proximal EGP of the left tibiotarsi of 42-day-old broilers in four experimental groups reared under two different environmental conditions and fed different experimental diets (*n* = 8/group). The parameters were evaluated as binary: altered (yes) or unaltered (no) EGP and normal (yes) and reduced (no) blood supply. LP/LA, basal diet according to the NRC, no supplementation; HP/LA, +5% linseed oil and NRC; LP/HA, basal diet according to Aviagen + 200 IU dl-α-tocopheryl acetate + 250 mg vitamin C + 0.15 mg Se/kg feed; HP/HA: +5% linseed oil and Aviagen + 200 IU dl-α-tocopheryl acetate + 250 mg vitamin C + 0.15 mg Se/kg feed; TN, thermoneutral environment; HS, heat stress environment.

The results of the blood supply assessment in the metaphysis are also shown in **Figure 3**. Because the expected frequencies in the experimental groups were less than 5 (chi-square test), a statistical comparison between the frequencies of the 8 groups was not applicable. The supply was reduced in 87.5% of samples in the HS HP/LA group and 75% in the TN HP/LA group, followed by 37.5% of samples in the HS LP/LA group and 25% in the TN LP/LA group. In the HA groups, the supply was reduced in 25% of samples in the HS LP/HA group and in 12.5% of samples in both HP/HA groups, with no change observed in the TN LP/HA group (**Figure 4**).

**Figure 4:**
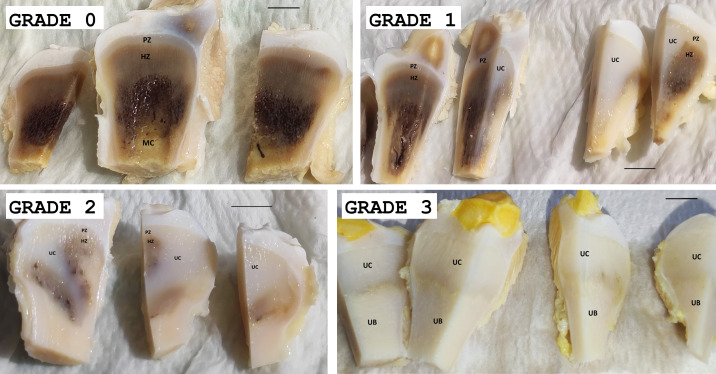
Macroscopic evaluation of the blood supply in the proximal extremity of the left tibiotarsi of 42-day-old broilers in which the EGP structure was not altered. **(A)** Normal blood supply; **(B)** reduced blood supply. Scale= 4 mm.

When the samples were grouped according to the fixed factors (environment, PUFA or antioxidants) into two experimental groups, the expected frequencies in the experimental groups were greater than 5. The chi-square test revealed a significant association between antioxidant supplementation and whether EGP morphology was macroscopically altered (χ2 (1) = 31.352) and blood supply was reduced (χ2 (1) = 13.576). On the basis of the odds ratio, the probability of changes in the EGP occurring was 45 times greater in the LA-fed group than in the HA-fed group. The odds of decreased blood supply were 9.18 times greater in the LA-fed group than in the HA-fed group. A correlation was also found between the PUFA content in the feed and blood supply, i.e., χ2 (1) = 4.433, and the probability of decreased blood supply was 3.15 times greater in the experimental groups given feed with higher PUFA content than in the groups given feed with lower PUFA content. No associations were found between changes in proximal extremity bone tissue and different environmental conditions.

### Geometry of the right tibiotarsus

The length, mass and volume of the tibiotarsus were influenced by the environment, PUFA content and antioxidant supplementation. However, interactions between these factors were observed for length but not for mass or volume (**Table 4**). The bone mass, volume, length and maximum width decreased in broilers maintained under HS conditions and in broilers that were not given antioxidant supplements, i.e., the LP/LA HP/LA. Increased dietary PUFA levels resulted in decreased bone mass, length and volume. Interactions between high dietary PUFA and HS were also observed for relative bone mass, as it increased in the HS HP/LA and TN HP/LA groups (**Table 4**), while HS treatment alone had no effect. Differences in cortical thickness were not observed between the experimental groups (**Table 4**).

**Table 4:** Tibiotarsus geometry and histomorphometry of the EGP zone of 42-week-old broilers.

	Environment									*P* value			
	TN				HS				SEM	E	F	A	E*F*A
Parameter/diet^1^	LP/LA	HP/LA	LP/HA	HP/HA	LP/LA	HP/LA	LP/HA	HP/HA					
Bone geometry (*n* = 12/group)													
Mass, g	22.49 ^abc^	19.91 ^cd^	24.35 ^ab^	24.49 ^a^	20.04 ^cd^	17.97 ^d^	22.85 ^abc^	21.30 ^bc^	0.71	**<0.0001**	**0.0035**	**<0.0001**	0.3915
Relative mass, %	0.87 ^bc^	1.01 ^ab^	0.78 ^c^	0.80 ^c^	0.85 ^bc^	1.13 ^a^	0.80 ^c^	0.82 ^c^	0.04	0.1842	**<0.0001**	**<0.0001**	**0.0103**
Length, mm	102.3 ^b^	96.1 ^c^	110.7 ^a^	109.9 ^a^	102.3 ^b^	88.6 ^d^	108.4 ^ab^	106.9 ^ab^	1.4	**0.0018**	**<0.0001**	**<0.0001**	**<0.0001**
Max width, mm	9.73	9.44	10.15	10.03	9.27	9.27	9.77	9.24	0.25	**0.0110**	0.1829	**0.0377**	0.7459
Volume, ml	20.3 ^abc^	18.7 ^cd^	22.0 ^a^	21.5 ^ab^	18.5 ^cd^	17.1	20.7 ^abc^	18.9 ^bcd^	0.6	**0.0001**	**0.0040**	**<0.0001**	0.8582
Cortical thickness, mm	3.80	3.61	3.77	3.58	3.51	3.78	3.63	3.35	0.21	0.4090	0.4998	0.5274	0.6588
EGP histomorphometry (*n* = 8/group)													
RZ thickness, µm	190	161	195	187	190	171	167	196	19.16	0.8448	0.4897	0.5588	0.6620
PZ thickness, µm	1036 ^ab^	870 ^ab^	656 ^b^	893 ^ab^	1243 ^a^	1303 ^a^	1010 ^ab^	1021 ^ab^	102.61	**0.0005**	0.6453	0.0048	0.4651
^2^HZ thickness, µm	510 ^ab^	579 ^ab^	735 ^a^	436 ^ab^	389 ^ab^	712 ^ab^	405 ^ab^	349 ^b^	106.02	0.2160	0.8709	0.4675	0.0288

^1^ Nomenclature of the experimental diets per experimental group: LP/LA, no supplementation with an additional fat source, basal diet according to the NRC; HP/LA, basal diet +5% linseed oil and NRC; LP/HA, no supplementation with an additional fat source, Aviagen + 200 IU dl-α-tocopheryl acetate + 250 mg vitamin C + 0.15 mg Se/kg feed; HP/HA, basal diet +5% linseed oil and Aviagen + 200 IU dl-α-tocopheryl acetate + 250 mg vitamin C + 0.15 mg Se/kg feed. ^a–d^ Within a row, values with no common superscripts differ significantly (*P* < 0.05). The statistical significance of differences between groups was analyzed using three-way ANOVA.

E, environment; F, fat; A, antioxidants; TN, thermoneutral environment; HS, heat stress environment; EGP, epiphyseal growth plate; RZ, zone of reserve cartilage; PZ, zone of proliferation; HZ, zone of hypertrophy (^2^ values from unmodified macroscopic samples included); LSMEANS - least square means.

The values are presented as LSMEANS (based on 4 birds per replicate and 3 replicates per dietary treatment), and variability is presented as the SEM. The number of samples used is given in parentheses.

### Histomorphology of the proximal EGP of the left tibiotarsus

Longitudinal sections of the tibiotarsi, subjected to various staining protocols, revealed EGP structures that were typical of birds (longer cell columns, randomly oriented proliferative and hypertrophic chondrocytes, and larger and numerous metaphyseal blood vessels, especially in the HZ, penetrating the PZ and RZ). In general, the RZ accounted for 12% of the plate, the PZ accounted for 60%, and the HZ accounted for 28% of the unchanged samples. Representative images of the proximal EGP of the tibiotarsus (Masson–Goldner staining) are shown in **Figures 5A–C**.

**Figure 5:**
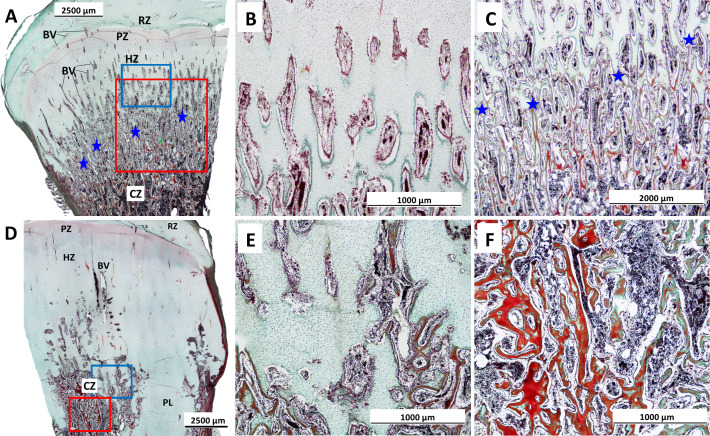
Micrographs of longitudinal sections of the proximal EGP in the tibiotarsus of 42-day-old broilers. **(A)** An overview micrograph of a typical plate structure with randomly oriented cell columns of chondrocytes in the proliferative zone (PZ) and hypertrophy zone (HZ) and larger and visible metaphyseal blood channels (BV) penetrating from the diaphysis into the PZ and the reserve zone (RZ). High-magnification micrographs marked with rectangles (**(B)**; blue square) show numerous blood channels with erythrocytes (dark red) with nuclei (black); (**(C)**; red square) and a clear calcification line of the cartilage matrix (blue asterisks; also in **(A)**; CZ - calcified cartilage in the ossification zone. **(D)** Micrograph of a sample from an animal with macroscopically visible changes in the architecture of the EGP and metaphysis, grade 3. Except the reserve zone (RZ), the plate zones are insufficiently developed, with sparse blood channels (BV), an enlarged proliferative zone (PZ) and a hypertrophic zone (HZ); the HZ intertwines with the calcified cartilage in the ossification zone (CZ), forming a nonmineralized and unvascularized compact mass (PL) and an enlarged zone of resorption of a dense meshwork of bone spicules. (**(E)**; blue square) Sparse blood channels in the HZ intertwined with the calcified cartilage matrix. (**(F)**; red square) The zone of resorption consisting of bone spicules of different sizes and orientations. Masson–Goldner staining; magnification 2x (**(A, C, D)** – large image), 4x **(B, E, F)**.

When a macroscopic change in the EGP architecture toward the medullary cavity was observed (grades 1–3 in **Figure 1**), changes in the thickness and morphology of certain zones were noted. The extent of the PZ, i.e., thickness, was less uniformly observable throughout the plate than it was in grade 0. Additionally, the blood vessels in the HZ were reduced or even absent. Compared with grade 0, the HZ was greatly enlarged and intermingled with the reduced and indistinct CZ, which overall formed a nonmineralized and unvascularized compact mass of chondrocytes extending into the metaphysis. In most samples, the resorption zone was greatly enlarged and structured into a dense meshwork of bone spicules of varying size, shape and orientation (not arranged longitudinally) and sparse connective tissue in the medullary cavity (**Figures 5D–F**). In some samples, the meshwork of bone spicules (the zone of resorption) was completely unmineralized, which was best observed following Masson–Goldner staining. The medullary cavity was smaller and farther from the proximal growth plate. Zones of EGP were not adequately developed in 40% of the samples (26 of 64).

AB staining was performed to facilitate morphometric measurements of the EGP zone thickness, which is shown in **Figure 6**. Assessment of the HZ thickness included only macroscopically unaltered samples (**Table 4**), and the area of the CZ in altered samples could not be properly assessed. None of the studied factors affected the chondrocytes of the CZ. Compared with the TN environment, the HS environment strongly increased the thickness of the PZ in the four groups, regardless of whether feed supplements or antioxidants were administered. On the other hand, antioxidants reduced the extent of the PZ, regardless of the housing conditions or PUFA content. In the HS groups without antioxidant supplementation (LP/LA and HP/LA), the PZ thickness was significantly greater than that in the LP/HA group, which was maintained in the TN environment (*p* = 0.0017 and 0.0023, respectively). The HZ thickness (without macroscopically altered samples) was lower in the HP/HA group maintained in the HS environment than in the LP/HA group maintained in the TN environment (*p* = 0.0410; **Table 4**).

**Figure 6:**
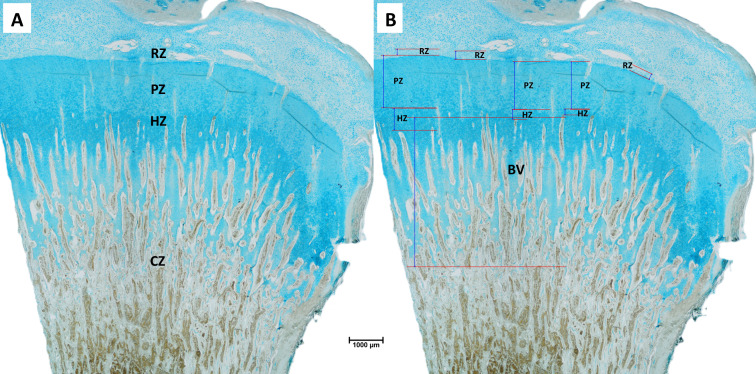
Thickness measurements of the EGP zones (longitudinal section of the proximal extremity of the tibiotarsus) of 42-day-old broilers; alcian blue staining, magnification 2x. **(A)** A typical plate structure with clearly visible distinct zones and metaphyseal blood channels (BV) penetrating from the diaphysis into the hypertrophic zone (HZ). **(B)** The thickness of each zone was measured at three locations in the growth plate cartilage. RZ, reserve zone; PZ, proliferative zone; CZ, calcified cartilage in the ossification zone.

### Biomechanical properties of the right tibiotarsus

A comparison of the biomechanical parameters of the right tibiotarsus (maximum load, displacement and flex modulus) that were determined using the three-point bending test revealed that, compared with the TN conditions, the HS conditions lowered the maximum load and flex modulus. A high proportion of PUFA in the diet decreased all three parameters, and compared with the LA group, the HA group exhibited a lower displacement and a higher maximum load (**Table 5**).

**Table 5:** Biomechanical properties of the tibiotarsus and bone content, including analysis of microelements of 42-day-old broilers (*n* = 12/group).

	Environment									*P* value			
	TN				HS				SEM	E	F	A	E*F*A
Parameter/Diet^1^	LP/LA	HP/LA	LP/HA	HP/HA	LP/LA	HP/LA	LP/HA	HP/HA					
Biomechanical property													
Max load, N	343 ^a^	284 ^ab^	314 ^ab^	320 ^ab^	252 ^bc^	168 ^c^	274 ^ab^	243 ^bc^	19	**<0.0001**	**0.0010**	0.1190	**0.0397**
Displacement, mm	4.02 ^bc^	4.76 ^a^	3.74 ^c^	3.61 ^c^	3.87 ^bc^	5.25 ^a^	3.64 ^c^	3.44 ^a^	0.20	0.9172	**0.0086**	**<0.0001**	**0.0098**
Flex modulus, MPa	739 ^abc^	562 ^c^	847 ^a^	833 ^a^	602 ^bc^	315 ^d^	794 ^ab^	724 ^abc^	50	**0.0003**	**0.0003**	**<0.0001**	**0.0391**
Bone analysis and mineral content													
Dry matter (g/kg)	206	211	239	249	212	186	235	241	16	0.5223	0.8970	**0.0024**	0.7927
Crude ash (g/kg DM)	408 ^bc^	389 ^c^	423 ^ab^	440 ^a^	389 ^c^	355 ^d^	419 ^ab^	432 ^ab^	7	**0.0006**	0.2312	**<.0001**	**0.0001**
Fat (g/kg)	19.1 ^b^	13.7 ^b^	55.5 ^a^	66.5 ^a^	19.7 ^b^	8.3 ^b^	56.6 ^a^	66.1 ^a^	3.8	0.6993	0.7369	**<0.0001**	**0.0179**
Ca (g/kg ash)	323 ^bc^	329 ^abc^	339 ^ab^	340 ^a^	332 ^abc^	317 ^c^	334 ^ab^	343 ^a^	4	0.5775	0.9434	**<.0001**	**0.0175**
P (g/kg ash)	164	163	170	166	169	165	168	168	2	0.1921	0.1905	0.0551	0.4874
Ca:P ratio	1.98 ^ab^	2.02 ^ab^	2.00 ^ab^	2.06 ^a^	1.97 ^ab^	1.92 ^b^	1.99 ^ab^	2.04 ^ab^	0.03	0.1036	0.2121	**0.0093**	0.1396
Mg (g/kg ash)	8.30 ^ab^	8.60 ^ab^	8.23 ^ab^	8.44 ^ab^	8.72 ^a^	9.13 ^a^	7.96^b^	8.15	0.24	0.5762	0.1014	**0.0040**	0.2545
Zn (mg/kg ash)	531	575	449	480	460	574	471	453	46	0.5490	0.1863	**0.0293**	0.6531
Fe (mg/kg ash)	325	314	345	401	355	332	403	360	24	0.3285	0.7604	**0.0082**	0.2872
Na (mg/kg ash)	24.6 ^bc^	26.3 ^ab^	19.5 ^cd^	18.8 ^d^	22.3 ^bcd^	30.1 ^a^	18.6 ^d^	20.2 ^cd^	1.3	0.0606	**0.0034**	**<.0001**	**0.0489**
K (mg/kg ash)	10.8^bc^	12.6 ^ab^	9.9 ^c^	9.8 ^c^	11.8 ^abc^	13.6 ^a^	9.4 ^c^	9.7 ^c^	0.6	0.3484	**0.0213**	**<.0001**	0.1003

^1^ Nomenclature of the experimental diets per experimental group: LP/LA, no supplementation with an additional fat source, basal diet according to the NRC; HP/LA, basal diet +5% linseed oil and NRC; LP/HA, no supplementation with an additional fat source, Aviagen + 200 IU dl-α-tocopheryl acetate + 250 mg vitamin C + 0.15 mg Se/kg feed; HP/HA, basal diet +5% linseed oil and Aviagen + 200 IU dl-α-tocopheryl acetate + 250 mg vitamin C + 0.15 mg Se/kg feed. ^a–d^ Within a row, values with no common superscripts differ significantly (*P* < 0.05). The statistical significance of differences between groups was analyzed using three-way ANOVA.

E, environment; F, fat; A, antioxidants; TN, thermoneutral environment; HS, heat stress environment; LSMEANS, least square mean.

The values are presented as LSMEANS (based on 4 birds per replicate and 3 replicates per dietary treatment), and variability is presented as the SEM.

### Bone mineral content of the right tibiotarsus

Compared with the TN conditions, the HS conditions decreased the crude ash content (4.01%) but not the contents of the other minerals. The addition of dietary n-3 PUFA increased the Na and K contents in the bone ash compared with those observed in the low dietary PUFA groups. Additionally, the inclusion of high antioxidant levels in the diet increased fat levels, Ca levels, Ca:P ratios and Fe levels in the bone ash and decreased ash, Mg, Zn, Na and K contents compared with those in the LA group (**Table 5**).

## Discussion

In this study, we investigated the combined effects of two stressors, i.e., cyclic HS (environmental) and dietary oxidative stress induced by high levels of n-3 PUFA, with two levels of antioxidants, namely, vitamin E, vitamin C and Se, on bone tissue and metabolism in fast-growing broilers. Tibiotarsi from 42-day-old male Ross 308 broilers were analyzed. The bone geometry (length, mass, width and volume) was decreased by the HS environment and high PUFA content but increased when high levels of antioxidants were administered. Histomorphological assessment of the proximal tibiotarsal extremity indicated the effects of antioxidants and PUFA on length growth. Additionally, architectural changes in the EGP resembling TD were observed in broilers housed in the HS environment, and these changes were mitigated to some extent by the administration of high antioxidant levels. Cyclic HS had a negative effect on bone strength and reduced the crude ash content in the tibiotarsus. Furthermore, high dietary antioxidant levels increased the bone ash content, fat content, Ca content and Ca:P ratio, whereas the Mg, Zn, K and P contents decreased. This study was part of a larger study investigating *in vivo* oxidative stress and the antioxidant status of broilers, with a focus on the effects on oxidative stress and antioxidant defense parameters as well as gut characteristics and fermentation activity (Rezar et al., 2024, Rezar et al., 2025). Similar to the conclusions of this study, cyclic HS and high levels of n-3 PUFA negatively affected broiler health status, causing damage to the intestinal morphological structure, whereas antioxidant supplementation mitigated the negative effects of the two stressors to some extent (Rezar et al., 2024). To our knowledge, this is the first comprehensive study to investigate the environmental or dietary effects of antioxidant supplementation on bone tissue and long bone biomechanics.

### Bone geometry and content

The reduction in length, mass and thus volume of the right tibiotarsus of broilers maintained in the HS environment and given high PUFA levels in their diets indicated the negative effects of cyclic HS and high levels of n-3 PUFA on long bone growth. In contrast, the improvement caused by supplementation with high levels of vitamin C, vitamin E, and Se (HA groups) mitigated these two negative stressors. Previous reports on body mass and the feed conversion ratio in these broilers revealed decreased body mass in the HP groups and increased mass in the HA groups on days 21 and 40, which led to an increased feed conversion ratio. In particular, on day 40, the mass of the HS HP/LA group was lower than that of all the HA groups under TN and HS conditions (Rezar et al., 2025) that we also observed in our study in right tibiotarsus. Similar results were reported in a study on HS and high PUFA or high antioxidant levels in which the animals were fed vitamin E and Zn as antioxidants, and 56-day-old female broilers were studied (Vakili et al., 2010). In our study, the abovementioned reduction in geometry, including relative bone mass in relation to animal size, was most evident in terms of bone length growth. Vakili et al (Vakili et al., 2010). reported that HS reduced the width growth of the tibiotarsus, despite the lack of change in cortical thickness, which was not observed in our study. In broilers that are exposed to HS, reduced growth and body mass are associated with reduced food intake, impaired digestion and impaired metabolism (Hosseini-Vashan et al., 2016; Horváth and Babinszky, 2018; Vandana et al., 2021), which could also contribute to differences in bone mass and size. The reduction in feed intake, digestion and metabolism of minerals associated with HS is also closely associated with the metabolism and mineralization of bone (Mir et al., 2018), which, together with respiratory hypoxia and changes in acid–base regulation, can affect mineralization and ultimately the chemical composition of bone (Arnett, 2007).

Since the functionality of bones depends on their geometry and composition, it is also important to note that, in our study, large changes in the mineral composition of the bones were also observed. Although the bone dry matter did not differ between the groups, the crude ash content was affected by HS, with the concentration of ash being lower in the animals exposed to HS than in those exposed to the TN environment, which is consistent with the findings of other experiments in broilers (Vakili et al., 2010; Hosseini-Vashan et al., 2016; Rocchi et al., 2022). This difference could be a consequence of the delayed maturity and growth of the animals (Rath et al., 2000), as the animals and bones in the HS groups were smaller than those in the TN groups were. In addition, the Ca and Fe contents and the Ca:P ratio increased in the HA groups, whereas the concentrations of ash, Mg, Zn, Na and K decreased. The nutrient composition of the HA groups apparently allowed for optimal bone growth independent of heat and n-3 PUFA stress. Therefore, the suboptimal growth of bone in the LA and HS groups also indirectly affected the contents of other minerals, as the concentrations of P and Ca were lower, with a bone ash proportion of approximately 95% (Rath et al., 2000).

The latter could also be confirmed by analyzing markers of bone turnover, as the HA groups had higher levels of iCa and lower levels of alkaline phosphatase than the TN groups did (Rezar et al., 2025). In addition to the differences in inorganic matter content, another notable difference between the HA and LA groups was the difference in fat content in the tibiotarsus. The bones of the animals in the HA groups consistently exhibited greater fat contents, which could indicate differences in energy metabolism and/or bone marrow adipose tissue growth and development between the HA and LA groups. These differences in bone could also be responsible for other changes in bone properties, as adipose tissue is responsible for local endocrine functions and thus for mineralization and other processes in bone (Moscatelli et al., 2024).

### Bone biomechanics

The biomechanical properties of bones are important indicators of bone functionality. In the present study, HS significantly reduced bone strength, as shown by the maximum load and flex modulus, whereas n-3 PUFA further reduced bone strength. On the other hand, increased intake of antioxidants and minerals (HA groups) contributed to improved biomechanical properties of bone. Both stressors exerted synergistic negative effects; the group that was maintained under HS conditions and supplemented with high n-3 PUFA and low antioxidant contents was the most negatively affected. It is difficult to predict whether the effects of HS are direct or indirect, as during cyclic HS, feed intake and the absorption of minerals and other nutrients are lower, whereas urinary and fecal mineral excretion are greater (Belay et al., 1992; Mir et al., 2018). Therefore, greater dietary supplementation with minerals and vitamins could be beneficial, as confirmed by our experiment. These findings are partly consistent with results observed in female broilers (Vakili et al., 2010), where the effects of HS were negative and those of high-antioxidant diets were positive, although n-3 PUFA (2% canola oil + 3% fish oil) improved the breaking strength of the bones. It is known that there is an optimal concentration of n-3 PUFA in diets; n-3 PUFA have positive effects at low doses but negative effects at high doses (El-Sayed and Ibrahim, 2017). The addition of n-3 PUFA had negative effects in the present study, presumably due to the induction of oxidative stress (Rezar et al., 2025).

### Bone morphology

On the other hand, morphological assessment of the left proximal tibiotarsal extremity indicated that the differences were influenced by antioxidant and/or PUFA levels but not by HS. The degree of macroscopic change in EGP architecture and the extent of the affected area was related mainly to lower levels of antioxidant supplementation, as little or no change was observed in the HA groups. Additional negative effects were induced by high levels of n-3 PUFA. Similarly, the metaphyseal blood supply, which is necessary for the physiological ossification process in the EGP and metaphysis (Huang et al., 2019; Jahejo et al., 2020), was reduced in the LA groups, and high levels of PUFA further hindered the development of blood vessels in the EGP. Altered tibiotarsus, undeveloped and hierarchically disrupted EGP zones lacked blood vessels but had an unmineralized and unvascularized mass of connective tissue in the HZ and CZ and/or unstructured, even unmineralized bone spicules (macroscopically seen as poor blood supply). These changes in the proximal epiphysis distanced the medullary cavity toward the diaphysis (macroscopically seen as a reduced medullary cavity).

The abovementioned macroscopic and microscopic changes in the epiphysis and metaphysis of the tibiotarsus can be classified as TD, which is a skeletal abnormality that is common among fast-growing broiler breeds (Leach and Gay, 1987; Huang et al., 2019; Jahejo et al., 2020). This abnormal skeletal development is thought to arise from a series of metabolic and structural changes in which condensed autolytic hypertrophic chondrocytes are unable to maintain or resorb the matrix, resulting in inadequate calcification. The inhibition of the penetration of the blood capillary network and the formation of vascular tunnels in the metaphysis lead to delayed transport of dead chondrocytes through the blood vessels. Since angiogenesis is necessary for chondrocyte hypertrophy and growth plate remodeling, failure of bone vascularization in the epiphysis during bone formation impairs endochondral development, i.e., length growth, and eventually leads to EGP lesions known as TD (Leach and Gay, 1987; Huang et al., 2019; Jahejo et al., 2020). The inhibited removal of apoptotic chondrocytes over time can lead to the accumulation of cartilage containing nonviable chondrocytes, resulting in elongation of the growth plate and preventing further ossification. Several studies have shown that TD is induced by impaired angiogenesis caused by various chemicals, such as pesticides (Rath et al., 2007a, Rath et al., 2007b), and heat shock protein 90 activity (Genin et al., 2008; Herzog et al., 2011). In contrast, high-altitude-induced hypoxia has negative effects on the growth of broilers but, surprisingly, has positive effects on the development of blood vessels in the tibial EGP (Huang et al., 2017). Tetramethylpyrazine, which is an ingredient of Chinese medicines, has angiogenic effects on thiram-induced TD (Mehmood et al., 2018), and high concentrations of vitamin D3 in the diet appear to prevent TD in broilers (Whitehead et al., 2004).

Dietary vitamin E has been described as an agent that potentially protects bones in animals of both sexes because of its antiosteoporotic properties, as it stimulates trabecular bone formation, i.e., the development and thickness of the EGP (Xu et al., 1995; Wong et al., 2019). Similar results in which high levels of antioxidants (vitamin E, vitamin C and Zn) attenuate the negative effects of HS and PUFA have been reported in older broilers (Vakili et al., 2010). In a more recent study, Calik et al. (2022) reported that HS had no effect on bone mineral content or bone mineral density; however, antioxidants increased both bone parameters in both environments. Supplementation with n-3 PUFA can influence bone remodeling and lead to bone loss (Wauquier et al., 2009); at high concentrations, n-3 PUFA (2% canola oil + 3% fish oil) reduced the strength and size of tibiotarsi in female broilers (Vakili et al., 2010). On the other hand, a positive effect of dietary supplementation with n-3 PUFA on bone formation and morphological characteristics was observed in young rats and broiler offspring when included in the maternal diet as well as a protective effect on bone mineral loss in ovariectomized rats (Watkins et al., 2000, Watkins et al., 2003; Tompkins et al., 2022). In rats, n-3 PUFA promote osteoblast function (Watkins et al., 2003) and inhibit osteoclastogenesis and thus bone mass loss (Sun et al., 2003; Kajarabille et al., 2013). Our results suggest that high levels of n-3 PUFA *per se* do not affect osteoblasts during appositional growth but do affect chondrocytes during endochondral bone development. The increased thickness of the PZ in broilers exposed to HS indicates an impaired ossification process in the EGP and disrupted mineralization of the tissue (Huang et al., 2019). It could be hypothesized that the abovementioned metabolic and structural changes in the EGP that were observed in our study and classified as TD were related to dietary nutrients (antioxidants and/or PUFA) rather than to environmental changes. However, we concluded that the growth of long bones, such as the tibiotarsus (length and width), was affected by PUFA and HS in this study. High levels of antioxidants appeared to alleviate, to some extent, the negative effects of both stressors on the occurrence of TD and long bone growth.

The aim of our study was to investigate these stressors in an experimental setting. High levels of PUFA supplementation were applied to induce oxidative stress. Notably, such levels were not intended for direct use in commercial chicken production, which could be a limitation of the study.

## Conclusions

Our results on the combined effects of HS, dietary PUFA and various antioxidants on fast-growing broilers suggest that cyclic HS and the administration of PUFA affect long bone length and width growth and mineralization, consequently affecting bone strength. The knowledge acquired regarding these stressors under experimental conditions may have potential applications in chicken production. The inclusion of high levels of n-3 PUFA in the diet indicated greater histopathological changes in the EGP architecture, whereas high levels of antioxidants mitigated these effects and facilitated bone growth and strength. These findings suggest that high levels of antioxidants may contribute to maintaining bone health and functionality, particularly under heat stress and high dietary n-3 PUFA conditions. The findings of this comprehensive study on bone tissue represent a step forward in the development of nutritional strategies to mitigate HS in fast-growing broiler production.

## Data Availability

The raw data supporting the conclusions of this article will be made available by the authors, without undue reservation.
